# Patients’ decision to contribute to a biobank in the light of the patient-recruiter relationship—a qualitative study of broad consent in a hospital setting

**DOI:** 10.1007/s12687-020-00479-z

**Published:** 2020-08-10

**Authors:** Francesca Bosisio, Gaia Barazzetti, Daria Koutaissoff, Brenda Spencer

**Affiliations:** 1 Institute of Humanities in Medicine (IHM), Lausanne University Hospital and Lausanne University, Lausanne, Switzerland; 2 Le ColLaboratoire, Unité de recherche-action, participative et collaborative, Lausanne University, Amphipôle, UNIL Sorge, CH-1015 Lausanne, Switzerland; 3 Institute of Social and Preventive Medicine, Lausanne University Hospital and Lausanne University, Lausanne, Switzerland

**Keywords:** Broad consent, Biobank, Genomic research, Health data, Communication in healthcare, Shared decision-making, Trust

## Abstract

Findings from recent studies show that the relationship developed with the particular researcher asking for broad consent plays an important role in the participant’s willingness to give consent. Interviews and focus groups were conducted in order to present a description and analysis of meetings in which broad consent took place and to examine the role of recruiters in the patients’ decision-making and in building trust in the Lausanne University Hospital Institutional Biobank (BIL). Our findings suggest that patient broad consent to biobanking is strongly related to its setting. BIL recruiters’ were aware of their role as ambassadors of the BIL and their responsibility towards patients. Patient interviewees were sensitive to the quality of the information delivered, the timing of the consent request and the recruiters’ attitudes and behaviours, including the presence of the white coat. Participating in the BIL also seemed to reinforce the patient’s self-esteem and perceived efficacy, particularly since they are themselves ill and inactive when requested to participate. Recruiters and participants report that participation may be motivated by fundamental (existential) goals. Organisational factors also affected recruiters’ activity and the broad consent procedure raising several ethical issues. This qualitative study suggests that biobanking based on information-based models of decision-making might need to be re-evaluated in order to improve broad consent. Our findings have implications for the practice of broad consent and patient autonomy, as well as for the recruiters’ role and training.

## Introduction

The last decades saw the proliferation of biobanks in many countries. In these biobanks, biomaterials and data are stored prospectively for the purpose of future research that is not yet defined and cannot be anticipated at the time of collection. Moreover, these collections of biological samples and medical records can be linked, manipulated and continually added to over time and can be accessed by a number of different users for various research purposes. In consideration of this uncertainty, several scholars in the field of biobanking have proposed the adoption of ‘broad consent’ for future use of samples and data instead of the conventional narrow consent for specific research uses (Elger and Caplan 2006; Hansson et al. 2006; Helgesson 2012; Otlowski 2009; Sheehan 2011; Steinsbekk et al. 2013). The rationale behind this concept is that in addition to other safeguards, such as the approval of all future projects by a research ethics committee, people can make a reasonably informed decision provided they are given relevant information about the prospective use of their samples and data. As a result, current debate about broad consent focuses on what kind of information counts as ‘relevant’ and should be included in consent forms (Grady 2015; Strech et al. 2016).

However, scientific literature in the field of consent highlights the limits of information-based models of consent, particularly since obtaining consent is a practice that remains irreducibly ‘opaque’ with respect to the patient’s actual understanding of the information and the reasons on the basis of which the patient makes a decision (O’Neill 2003). Therefore, understanding informed consent as a form of respect for autonomy reveals a tendency to simplify what constitutes autonomous deliberation and the respect it is due. A simplification that, as O’Neill points out, is undoubtedly specific to healthcare and biomedical research contexts, in which the idea is becoming increasingly widespread that the patient is a consumer of services or a provider of health data and biological samples, rather than an agent who decides autonomously (O’Neill 2003). For this reason, several authors have called on alternative models able to acknowledge other ways of knowing and deciding (Ezrahi 2004; Hermann et al. 2017).

Broad consent may serve within this context as a magnifying glass to address the limits of information-based consent procedures. Indeed, in broad consent, essential elements of informed consent, such as information about the nature of future research and its risks and benefits for the participant, their families or the society, are lacking. Consent to biobanking moreover is not contingent on a medical procedure but can possibly happen in a care setting. Several scholars in the field of biobanking for genomic research have indeed suggested that trust may substitute for lack of appropriate information (Hawkins and O’Doherty 2010; McNamara 2007). Interestingly, findings from a recent study show that the relationship developed with the particular researcher asking for broad consent plays an important role in participant willingness to give consent (Kelly et al. 2015).

The Lausanne Institutional Biobank (BIL, presently called CHUV biobank—Biobanque génomique du CHUV) was established in 2013 at the Lausanne University Hospital (CHUV) in order to collect patients’ biomedical data and samples for prospective genomic research, with a view to developing innovative therapeutics and biomarkers and advancing the field of personalized medicine (Mooser and Currat 2014). People accepting to participate in the BIL gave permission for coded or anonymous use in research of their biological material (blood or leftover tissues) and medical and genomic data. Patients could also indicate whether they would like to be re-contacted to receive individual clinically relevant research results. Between January 1, 2013 and June 31, 2015, 79% of the 25,721 people asked to sign the BIL broad consent agreed to coded participation and 5.5% accepted the anonymous form; 15% declined to participate in the BIL (Bochud et al. 2017).

Since its establishment, the BIL has deployed constant efforts in order to improve its broad consent procedure (see box 1 in Barazzetti et al. 2020 for an overview). At the time of this study (2013–2014), two broad consent procedures were used at the same time. People admitted for a planned procedure—either as out- or in-patients—received information about the biobank and consent information by post mail around 2 weeks before their appointment. In addition, the BIL employed a team of recruiters (made up of nurses, medical assistants and research personnel), specifically trained to ask for patients’ broad consent. Usually, recruiters met only patients that were hospitalized following an emergency admission or patients that were undergoing a planned procedure—either as in- or out-patients—but who did not send back their consent form before admission. These meetings were always held at the hospital. The recruiters’ assignment during their meeting with patients was to promote an autonomous decision about participating in the biobank; it was not to persuade people to participate. In order to ensure adequate information and neutrality, recruiters were trained and provided with a standard interview guide that gave essential information about BIL goals and procedures and the implications of anonymous or coded participation.

In this paper, we present the results of a qualitative study of broad consent within a hospital-based biobank. With a view to capturing the intersubjective and contingent nature of the decision to participate in the BIL, we describe and analyse meetings in which broad consent happened at a specific point in time and examine the role of recruiters in the patients’ decisions to subscribe to broad consent and in building trust in the BIL.

## Material and methods

The results presented in this paper were derived from data collected through interviews and focus groups taking place between October 2013 and November 2014 at the Lausanne University Hospital. Data collection was conducted following IRB approval.

### The research team

The research team was composed of the authors, all women. All the researchers are trained in qualitative research. The tasks related to data collection, data analysis and discussion of results for publications were divided among the members of the research team as follows (Table 1):

**Table 1:** Division of tasks

Authors:	FB	GB	DK	BS
PI and bioethics expertise		X		
Project coordination		X		
Methodological supervision				X
Recruitment	X	X		
Conducting the interviews	X		X	
Parallel coding of the interviews	X		X	
Analysis of the interviews	X	X	X	X
Coding of the interviews			X	
Conducting the focus groups	X	X		
Parallel coding of the focus groups	X	X		
Analysis of the focus groups	X	X		
Discussion of results in preparing publications	X	X		X
Writing and revision of articles	X	X		X

Before the interviews and the focus groups, the researchers had no relationship with the participants. The researchers introduced themselves to the participants (first and family name, affiliations and training) during the first meeting. The purpose of the study was also explained to the participants during the first meeting.

### Study design

In this study, interviews and focus groups were conducted concomitantly in order to explore the practice of broad consent at the Lausanne University Hospital. Interview guides were developed based on a preliminary literature review and the material made available by the BIL managers. Themes addressed in interviews and focus groups are listed in Table 2.

**Table 2:** Themes addressed during interviews and focus groups

Interviews with patients	Understanding of the biobank’s functioning and purpose Understanding of broad consent Information provided by the biobank recruiter Perception of biological material Data confidentiality Decision-making process Return of individual research results Personal expectations and suggestions
Focus groups	Contextual factors relevant to broad consent practice Perception of recruiter’s role Patient’s decision-making process Perception of the biobank mission

### Patient selection and method of approach

A purposive sampling framework for the interview was drawn up based on patient characteristics: decision regarding participation in the BIL (coded participation, anonymous participation or refusal to participate), sex, age and mode of admission to Lausanne University Hospital (emergency or planned procedure). This sampling strategy was consistent with the findings of Bochud et al. (2017) that highlighted that age, sex and mode of admission in the BIL induced significant differences in whether patients accepted to participate.

Potential participants in our study were patients pre-screened to meet a recruiter, either because they were admitted as an emergency or had a planned procedure and did not send back their broad consent form before admission. A nurse practitioner met people eligible for our study some time after the recruiter’s visit in order to establish if they were interested to participate in an interview about their perceptions of broad consent for the BIL. If so, the nurse transferred the patient’s details to FB or DK, who were in charge of calling the patient to set up a meeting, explaining and presenting the consent form to the patient and, if the patient consented, carrying out the interview.

Focus group participants were recruited by e-mail. In order to increase participation, focus groups were conducted during a normal working day with the permission of BIL managers. Participation was voluntary, and the identity of the recruiters participating in the focus group was not disclosed to BIL managers by the researchers. FB and GB sent the written information and the consent form to the participants 1 week before the meeting. The first 10 min of each focus group was dedicated to questions and signing the consent forms.

### Sample size and composition

All patients eligible to participate accepted to be interviewed. We carried out 22 semi-structured interviews with patients who accepted either coded participation (*N* = 12), anonymous participation (*N* = 4) or refused to participate in the BIL (*N* = 6). Table 3 in the “Results” section summarizes the interviewees’ characteristics.

**Table 3:** Interviewees’ characteristics

	*N* = 22	Proportion
Women	6	27%
Men	16	73%
Age		
36 to 50 years old	7	32%
51 to 65 years old	8	36%
More than 66 years old	7	32%
Participation to the BIL:		
Accept	16	73%
Refuse	6	27%
Consent asked:		
At the hospital, after an emergency admission	7	32%
At the hospital, after a planned admission	10	45%
During the pre-hospital surgery consultation	3	14%
By post mail before a consultation or an elective procedure	2	9%

Eleven of the thirteen recruiters accepted to participate in a focus group. One was absent the day of the focus group. The ten participants were divided into two groups. The first (FG1) was composed of 4 newly trained recruiters that had worked at the BIL for less than 3 months. The second (FG2) consisted of 6 people that had worked at the BIL for more than a year. No table with focus group participants’ is provided in order to grant full depersonalisation of the qualitative data collected. Indeed, most of the 13 recruiters that accepted to participate in this research are still employed.

### Setting

The individual interviews were conducted in a place chosen by the participant. FB and DK met people at their home or at the hospital. One interview involved both the patient and his wife. Patients were otherwise alone with the interviewer. No table with focus group participants’ is provided in order to grant full depersonalisation of the qualitative data collected. Indeed, most of the 13 recruiters that accepted to participate to this research are still in employment.

The focus groups brought together the participants in a quiet conference room in the hospital building and were conducted by two researchers: a moderator (FB), leading conversations on the basis of a pre-established interview grid, and an observer (GB), noting participants’ non-verbal expressions.

### Data recording and duration

Interviews lasted between 32 min and 1 h 6 min and the focus groups between 1 h 37 min and 1 h 48. With the permission of the participants, all focus groups and interviews were audio recorded and transcribed verbatim for analysis. The transcriptions were not returned to the participants.

### Data analysis

Thematic analysis was carried out in order to identify codes and derive themes and categories from the data (Braun & Clark 2006). The first five interviews were coded by DK and FB separately in order to check the reliability of the codes. A similar procedure was adopted by FB and GB with the first focus group. Inconsistencies were discussed with reference to the raw data until a consensus was achieved. The interview thematic analysis was assisted by MaxQDA and the focus groups by RQDA. The themes emerging from the analysis are explained in the findings.

Table grouping codes and themes were discussed within the research team. Findings from interviews and focus groups were compared in order to check the consistency of the resulting categorisation and to increase methodological reliability (Flick 1992).

## Results

### Interviewees’ characteristics

Seventeen of the 22 patients were asked for broad consent whilst hospitalized. This rate is consistent with Bochud et al. (2017) and with the broad consent procedure in place at the time of our study.

### Findings

Requesting and giving broad consent to participation in a BIL in a hospital setting involves several challenges for recruiters and participants in order to meet the conditions of informed consent. Although interdependent, for the purpose of our description, we present them in accordance with the main themes issued from our thematic analysis: (1) assessing the patient’s decision-making capacity, (2) providing information and (3) role of recruiters in patient decision-making. Within this last theme, we discuss (3.1) acting as spokespersons of the BIL and (3.2) connecting with patients. Participants’ narratives and notes are translated from French to English.

#### Assessing decision-making capacity

Requesting broad consent involves assessing patient decision-making capacity. Recruiters were not specifically trained to assess decision-making capacity and, moreover, were meeting patients for the first, and possibly last, time. Therefore, when possible, they asked nurses and physicians for information about the patients’ decision-making capacity before meeting him or her (focus group 1). When there was no information available, their assessment was basic:

If we have the feeling that the patient did not understand… we do not take the risk [to make him sign a consent form]

Focus group 1

And the patient instantly said: “I am not interested, goodbye”. And I said: “There’s no problem, Sir”. I left and put “non eligible” on his form, to protect him and my colleagues.

Focus group 1

Their concern is at the same time legal – to avoid abusing someone’s weakness (focus group 1) – and moral. This moral issue is more apparent when consent is requested from teenagers:

My hierarchy says that teenagers older than 16 can give consent, but I feel it is not… morally sound. I am myself a mother of teenagers and would be angry if I discovered that someone had made them sign a consent without asking me. (…) I think it is important to involve the parents.

Focus group 1

#### Providing information

The information to be provided when requesting broad consent was well standardized within the BIL. The data we collected do not allow us to assess whether the standard procedure to inform patients was respected. Recruiters’ reports, however, show that it was often difficult to have enough privacy and time to present this information and answer questions:

So, I opened the door and… “Oh, there are 5 people in the room!” and you are there and the physician is examining someone, and the nurse is drawing blood from someone else,… and when you start speaking with a patient, a lady comes in to give him an ECG. Well, this may have happened to you too… [assents in the group]

Focus group 2

The presence of others was a problem for recruiters: speaking in front of nurses, physicians and other patients puts them under a great pressure (FG2). They also felt this was a problem for patients. Indeed, there was a constant risk of interruption.

I have to request consent of pregnant women that come for a routine check-up before they go in for the check-up (…) With the fluctuations of the physician’s schedule, the physician can call the woman I am interviewing at any time.

Focus group 2

Well, some days it can be stressful. The patient may be in another room, or may be downstairs for examinations, or he may have been moved to the intermediary care unit. When they are in the intermediary care unit, they are lost to us.

Focus group 2

Sometimes I am presenting the consent and the physician comes in and asks me to stop and go out since he has to finish his visits. Of course, [caregivers] have priority.

Focus group 2

When patient interviewees were asked about the recruiter’s visit, Interviewees 1, 2, 8, 12, 18, 20 remembered that the meeting was very short but stated that recruiters offered them more time to think about their participation. They did however sign for consent straight away. Interviewees 5 and 10 felt that the meeting was very short. Interviewee 10 said that he quickly made his decision and did not need more time to think about it. Interviewee 19 and 23 asked for some time to think about their participation in the BIL, and a recruiter came back a few days later.

Some other interviewees were not satisfied with the timing of the visit. Interviewee 13 disapproved that the institution prioritized broad consent over his basic needs:

When the recruiter passed by… it was a moment when I was waiting for other information from the hospital. Information was important to me, about my recovery, the insurance, when I could go back home (…), and I did not have any follow up about all that. But suddenly someone had time to visit me and question me about the broad consent.

Interviewee 13

Interviewee 2, 3 and 27 also felt that the conditions in which the conversation took place did not feel right. Interestingly, the narratives of interviewees 3 and 27 confirm recruiters’ fears that conditions for decision-making are sometimes not met. Interviewee 3 said that, among other reasons, she refused to participate in the BIL because she did not want to engage in a conversation that her neighbour could hear. Interviewee 27 refused to participate in the BIL since, at the moment of the visit, he felt emotionally distressed and that he could not make such a decision.

Other factors, independent of the explanations provided by the BIL recruiter, influenced patients’ decisions to participate in the BIL. Most accepted based on a cost-benefit argument: they felt that biobanks and medical research are useful, whereas giving 100 ml of blood was not a significant issue (Interviewee 8). Participating also seems to reinforce patient’s self-esteem and perceived efficacy, particularly since they are themselves ill and inactive at the moment of the request to participate in the BIL:

I feel worthy… it is satisfying to tell myself that this can be useful to someone or to medical research in general.

Interviewee 1

Most people agreed to contribute to the BIL in order to feel useful and did not need more information than that supplied. Interestingly, most barely recalled the information given but were able to recall that samples of blood would be collected during the hospital stay. Only interviewees 3, 10, 13, 18 and 26 remembered that other tissues could be procured. Just one interviewee (1) mentioned that medical data could also be disclosed. Some patients had asked questions of the recruiter (interviewees 1, 2, 10, 16, 18, 24 and 28). Interviewee 3 looked for information in the internet and scientific literature, interviewee 13 spoke with colleagues and looked in the press, and interviewees 16, 20 and 21 visited the BIL website.

Patients refusing to participate or choosing to participate anonymously did so because they felt uncomfortable consenting to research which had yet to be defined (interviewees 3, 18, 25 and 27), contributing to pharmaceutical companies’ income (interviewee 26) or disclosing personal data potentially available for aims other than medical research (interviewees 13, 23, 24 and 27).

#### Role of the recruiters on patient’s decision-making

##### Acting as spokespersons for the BIL

Recruiters participating in the focus groups emphasised that they felt responsibility to represent the BIL and the hospital. During the focus group, Mr. A underlined that he was an ambassador of the BIL and a representative of the hospital institution: thus, he made efforts in order to be rigorous when presenting the biobank goals and the consent form. Likewise, Ms. E and Ms. D stressed they were the bricks that contributed to the construction of the BIL. Within this framework, they stressed the importance of being honest and trustworthy, in particular when explaining the issues of broad consent:

We don’t have the information [about potential future researches]… but we try to be honest about that. We discuss that… we say [to the participant] that we cannot give them details, but the Cantonal Research Ethics Committee will review and monitor the projects that will be performed in the future.

Focus group 1

Recruiters expressed very different perceptions of their role. Some felt their mission was to promote patients’ participation in the BIL: three participants used several times the metaphor of ‘selling’ broad consent during FG1. Other recruiters considered their role as being to promote conditions conducive to autonomous decision-making by the patient. Therefore, when the patient does not seem ready to decide, they cease the exchange:

A few days ago, I met someone who... it was the third time we came to see her and she wanted more information. She said: “I would like to look on the internet”. I said: “Maybe it is better that you say ‘no’ and then come back when you have made a decision because... it is better to say ‘no’ now so that you have time to make your own decision”. Because I understand that it is hard for them... it is a lot of information and it is intricate and for some patients it is... it is maybe better to take more time to make a decision.

Focus group 1

Interviewees’ accounts of meeting the recruiters tended to confirm that broad consent acceptance may rely on the perception formed of the recruiters as spokespersons of the BIL or the hospital.

Most interviewees had a positive perception of the encounter with the recruiter. Interviewee 9 said that the woman who came to see him took the time to go through the BIL brochure with him and answer his questions. She was nice and inspired trust:

I feel that if it is the nurse or the physician, it’s better. They are bound by ethics

Interviewee 9

This trust was not only inspired by people but also by institutions:

(Organ donation), I could do that, it’s something I could see myself doing – especially knowing that I could save someone, help someone, (…), but yes, yes, for me it’s part of the same impulse, the same, the same intention.

Interviewee 8

Interviewee 10 also perceived the recruiter as very professional: she answered all the questions and repeated that he could call her if he had additional questions. Interviewee 2 and other people thought that the recruiter was in fact a researcher and said:

I trust those people, people that make those studies.

Interviewer: That data will be used accordingly to what they said

Yes. In particular since we live in a country where things work pretty well, where there is no problem in that respect

Interviewee 2

Mistaking the recruiter for the researcher may be a problem. Ms. B remarks the impact of meeting the patient as a hospital representative:

The patient may not be able to say no. The white coat… a white coat is always…

Focus group 1

Even though the influence of the coat was not explicitly discussed during interviews, several interviewees express the fact that patients have no choice but to welcome the recruiters. Interviewee 2 said that the recruiter came when she was experiencing the acute phase of a bile duct infection and was not prepared for a discussion about research. Interviewee 3 said the recruiter visited him after lunch, when he normally takes a nap. He also explained that, as a patient of the hospital, he felt captive as he could not move away from his room. Interviewee 16 however stated that he did not feel pressured at all to make a decision:

The meeting was good. I felt the recruiter was open to all the questions I could have and… no, no, we took the necessary time to discuss… it was not a quick meeting, between other meetings.

Interviewee 16

Being a BIL or hospital spokesperson also seems to raise expectations regarding the recruiter’s status. Interviewee 13 had the impression that the recruiter underplayed the intrinsic risks of broad consent, that is, that employers, health insurances and the police could use information collected in the biobank to monitor or track someone. In his opinion, the recruiter was not prepared at all to answer such questions:

[I felt that] she was all… peace and love, so far so good. (…) She was kind and all, but she was not… “scientific”. I felt that she could not answer my questions so I did not ask them.

Interviewee 13

On the other hand, interviewee 3 highlighted that the recruiter who had visited her used many difficult words without explaining them. She thought that the recruiter was a student looking for participants for a thesis dissertation and so could not take this seriously.

##### Connecting with patients

Recruiters report that requesting broad consent of people they do not know requires that a connection be created with them. Several strategies are deployed to do that.

First, recruiters paid attention to clues regarding the patient’s educational level when engaging in the request of broad consent:

When we introduce ourselves to a patient we already see [patient’s education level] through the manner in which they ask the first question. In any case, I have to ask some questions about profession and educational level – university or training degrees – for statistical purposes. Thus, if I have a doubt, I ask them this information as an introduction.

Focus group 2

Second, recruiters report that sometimes, they spend time listening to patients’ stories and complaints. Indeed, in both focus groups, recruiters expressed consistently the opinion that some patients need this. More importantly, three recruiters reported during focus group 1 that the relationship they created with the patient was deeply satisfying and a powerful driver of their job motivation/satisfaction, compensating for the frustration sometimes procured:

With the staff, it’s not easy. With patients, on the other hand, it’s ok. At the beginning, I fear to bother patients with a request that would be the last of their problems. (…) but on the contrary, they generally welcome us and tell us that we do not bother them at all.

Focus group 1

For one recruiter in focus group 2, herself once a patient, it is necessary to connect with the patient to request their consent:

The staff… they don’t have any time to spend with the patient. Their mission is to monitor vital signs, as if the patient were a machine. I am sorry, but I felt like that. But we, to do our job properly, it’s not this side that we should care for. (…) We have to engage with the patient, be empathic.

Focus group 2

Another recruiter explains the necessity to connect with patients:

The nurse, she has something to give to the patient whilst we, we have something to ask of the patient.

Focus group 1

Some interviews tend to confirm that the recruiters’ ability to connect with them played a role in their decision. One interviewee said:

The discussion was honest and the lady was kind and dynamic. I think that my decision to consent [to the BIL] was influenced by that.

Interviewee 16

I feel worthy… it’s satisfying to tell myself that this can be useful to someone or to medical research in general.

Interviewee 18

Even though most participants (interviewees 8, 13, 18, 19, 23, 28, and 25) deny that the recruiter’s behaviour or their perception of him/her influenced their decision, an ability to connect with the potential participant and give an impression of competence may help patients decide whether to trust the overall BIL endeavour. This assumption is supported by narratives of good and bad experiences with the recruiter:

The lady that explained us the… the broad consent was a nurse. We felt we had all the time we needed to ask questions and discuss with her. I think it’s important to have a nurse or a physician in front of us for such decision.

Interviewee 20

So, [the recruiter] was not a scientist. She was very kind, very kind, and sensitive but (chuckles) she was not…

Interviewer: Convincing?

Yes… (…) I felt that I had someone in front of me that did not have the competence to answer my questions.

Interviewee 3

Interviewee 3 is, however, also aware that the circumstances in which the visit took place also had an impact on his decision not to participate in the BIL:

A recruiter came to see me soon after my surgery when I was expecting information about my insurance coverage and about my work leave. I was therefore a bit bothered that the hospital could send me someone to ask for my consent but not satisfy my requests to meet a social worker and a physician.

Interviewee 3

These results speak in favour of broad consent being a contingent decision that does not strictly depend on information giving. We discuss this assumption in the next chapter.

## Discussion

We acknowledge that the results presented above have certain limitations. Even though we selected interviewees in such a way as to take into account the variability in patients recruited to participate in the BIL, it is possible some aspects of variation escaped us owing to the small number of interviews conducted. The focus groups, on the other hand, involved the majority of the recruiters employed at the BIL at that time. Nevertheless, discussion guides were designed to focus on specific aspects of their activity and their interpretation of their meetings with patients, at which we were not present. For these reasons, any generalization of the results presented in this paper must be cautious.

Results have unveiled three important considerations in knowledge acquisition and subsequent competent autonomous decision-making to participate in the BIL: (1) the role of recruiters in patients’ willingness to participate or not in biobanks, (2) autonomy in biobanking and (3) fundamental goals in broad consent. We also discuss implications for practice and training.

### The role of recruiters in patients’ willingness to participate or not in biobanks

Most of the literature on consent and broad consent has focused on information and on barriers to contributing to biobanks (Grady 2015; Strech et al. 2016). Few articles have explored the researchers’ or recruiters’ role in supporting patient’s consent to participate in a biobank. Narratives and notes derived from the present study tend to suggest that recruiters may play a pivotal role.

In the very early stages of the focus groups, recruiters expressed the opinion that their attitude may count in the patient’s decision to participate in the BIL. Therefore, they endeavoured to act as ambassadors for the BIL and the hospital. The results of the interviews tend to confirm that patients share this perception of recruiters as institutional spokespersons and that this partially influences their decision about whether to participate. This is consistent with previous work in the field (Ahram et al. 2014; Facio et al. 2011; Kelly et al. 2015). Our results further this notion by suggesting that recruiters play an active and essential role in broad consent by rendering consent terms more comprehensible and, where possible, reducing uncertainty and increasing acceptability. Accordingly, focus groups underlie recruiters’ multiple roles and identities (Goffman 1959). The first is to appraise the patient’s competence to make a decision. The second is to act as mediators by adapting information to the patient, answering questions and, when necessary, connecting the patient with BIL heads for further explanation. The third is to be a compassionate listener. Those attitudes and the empathic way in which they are delivered may all together be conditions for the creation of a trusting relationship between the participant and the BIL. Many scholars (Hawkins and O’Doherty 2010; McNamara 2007; O’Neill 2004; Tutton et al. 2004) assume that trust is an essential determinant of participants’ decisions to contribute to biobanks. These data and the BIL’s high consent rates at the time of this research (Bochud et al. 2017) suggest that building a trust-relationship was an essential element of recruiters’ work.

These findings are reinforced by Whitley, Kanellopoulou and Kaye’s work (Whitley et al. 2012) emphasising that although researchers prefer broad consent models for pragmatic reasons, they feel awkward when explaining that the studies for which they are asking consent are not yet defined. The position of recruiters is thus ambiguous. On the one hand, they should represent the institution, with its organisational characteristics and research mission. On the other hand, they seem aware of the patients’ needs and interests and of the potential conflict with their role as spokespersons of the hospital. This conflict of role is usual in research in healthcare settings where staff may feel uneasy in their role as a researcher (Guillemin et al. 2017). Recruiters stated that their success in obtaining consent is dependent on constantly navigating between the interests of the institution and the patient.

In the next paragraph, we add elements to this argument and discuss the influence of the context on the recruiters’ work and potential implications for patient autonomy. In an article by Bochud et al. (2017) on the BIL consent rates between 2013 and 2015, the authors highlight that “Patients who received specific mailing prior to their admission were found to be better informed and ready for a deeper discussion on research when they met the recruiters”. Since the study reported here, the BIL decided to put an end to encounters between a trained recruiter and a patient. Patients that are admitted as an emergency receive information and consent form 14 days after their discharge. A hotline was also created to answer any questions. This new practice, designed to improve patient autonomy by providing sufficient time to inform about broad consent and biobanking, has nonetheless its own ethical issues. In particular, patients’ understanding and decision-making capacity are not assessed prior to signing the broad consent. In the light of these results and changes in BIL procedure, further research at the BIL should focus on what supports broad consent and trust building within this new endeavour.

### Autonomy in biobanking

The broad consent procedure used at the BIL at the time of the study reveals potential challenges and ethical issues. From an ethical point of view, it is worth asking how the intersubjective patient-recruiter relationship, explained as a necessity by several interviewees and focus group participants, influences the patient’s autonomy. As discussed above, recruiters’ influence on patients’ decisions may play out in different ways. Despite most recruiters stating that the BIL operational management does not pressure them to obtain patients’ consent or persuade patients to participate, this may occur implicitly through contextual factors. First, there is the white coat and the fact that several interviewees confused recruiters for nurses, physicians or researchers. Second, several interviewees stated that in the hospital context, when someone comes with a consent form, patients may not feel they have the option to refuse participation. Third, in consideration of the celebratory narratives associated with the high consent rates of the BIL (Aguzzi 2017; Bochud et al. 2017), obtaining consent may implicitly be construed as successful work and non-consent as a failure.

It is also worth highlighting that at the time of the study, 3 out of 4 people were asked while hospitalized to participate in the BIL. Issues in terms of ethics are twofold within this context.

Firstly, people that are hospitalized can be considered ‘captive’ in the hospital. Interviewee 3 highlighted this concern and was bothered by the recruiter’s visit since he was hoping to meet someone who could inform him about treatment and administrative issues with his health insurance. Interviewee 2 also stated that the timing of the visit was inappropriate. Recruiters also highlighted that they felt that the white coat they were wearing may be a source of confusion for patients. In Switzerland, the white coat, particularly in hospital settings, usually identifies physicians even though other persons, such as researchers, laboratory employees, psychologists or social workers, may wear it for their activities. Within this framework, patients might not realize that they are not in the presence of a physician and that the broad consent request is not tied with care. They might therefore not feel free or comfortable to refuse participation in the biobank. This assumption is supported by literature highlighting that the presence of a white coat tends to increase informed consent to research in DNA banking (Merz et al. 2002) and that authority—symbolized by the white coat—and trust are related factors that play a role in a patient’s decision regarding medical treatment (Brase and Richmond 2004).

Secondly, recruiters reported that assessing decision-making capacity presented challenges due to the general lack of criteria to assess patient’s capacity and the fact that the staff were not keen on sharing the necessary information about patients with recruiters. As a consequence, in an attempt to protect patients from potential abuse of their right to autonomy, recruiters tended to be conservative in their assessment of patient decision-making capacity and exclude people whom they doubted had the necessary capacity to consent to biobanking. This mechanism suggests an assessment of eligibility by adding supplementary criteria regarding the severity of patient’s symptoms, particularly the more severely ill (Hanson et al. 2014). Even though recruiters intended to protect the patients’ autonomy, it is worth asking whether such overprotective behaviour might not result in gatekeeping (Sharkey et al. 2010) and introduce bias in the sampling.

In the light of the results presented here and the changes in the BIL organisation presented above, future research on broad consent at the BIL could explore whether patients’ autonomy is supported or decreased through the changes made and how their decision-making capacity and understanding could be assessed within this framework. Such research would add to the critical literature on the influence of organisations on patient’s decision-making. Indeed, people’s decisions and professional assessment of patient understanding do not happen in a void, and scientific literature increasingly highlights the personal, contextual and relational factors of decision on health-related issues (Bosisio et al. 2013; Hermann et al. 2014, 2017; O’Neill 2004). It seems worthwhile within this context to elaborate indicators that go beyond consent rates and rather reflect patients’ satisfaction with the process, perceived autonomy and self-assessed understanding.

### Fundamental goals in broad consent

In a previous paper (Barazzetti et al. 2020), we argued that participation in the BIL may, to some extent, be understood as a gift to the institution. We would argue here that patients seem to refer to gift rationales as a way to convey their fundamental goals whilst deciding whether to participate or not to a biobank in the presence of a recruiter.

Consistent with several scholars’ observations, part of our findings suggest that the consent procedure follows guiding principles of shared decision-making (Delany 2007; Elwyn et al. 2012; Olufowote 2010) since it aims to provide information, offer options, promote deliberation, and eventually confer agency about further use—and reuse—of medical and genomic data and biological samples. An increasing body of literature on shared decision-making advocates that discussion on fundamental (or existential) goals (Vermunt et al. 2018) support patient decision-making about treatment options. Discussing such fundamental goal is particularly important in advanced decision-making having the aim of anticipating situations in which patients will not be able to decide for themselves.

Broad consent within the BIL can in a way be considered one of those advanced decisions. Participants in the BIL accepted to participate without knowing the nature and aim of future research on their data and biological material and waived their right to opt in or out of future research. Literature and data from past (Barazzetti et al. 2020) and present findings suggest that participants may refer to gift rationales in an attempt to convey the fundamental values and goals of their decision whether to participate. This assumption was not specifically addressed in our study but was spontaneously reported by recruiters and conveyed by patients during the interviews, suggesting that patients may feel the need to address such values.

In the light of these considerations, we advocate for fundamental goals to be explored during broad consent procedures as they may improve informed consent. In order to provide recruiters or researchers with tools to explore participants’ fundamental goals, their training may inspire from the training provided to clinicians.

## Conclusion

This qualitative study suggests that current conceptions of broad consent, based on information-based models of decision-making, might need to be re-evaluated, since decisions regarding biobank participation appear to be deeply contingent on their setting, to be influenced by relational factors and to depend on fundamental values such as trust in institutions and the quality of the relationship patients and recruiters establish during their meeting. Our findings have implications for the practice of broad consent and patient autonomy, as well as for the recruiters’ role and training.

## Data Availability

Interview and focus group transcription are available on demand. You can contact the corresponding author to obtain them.
